# Cardiac Involvement of Rosai-Dorfman Disease Causing an Aesthetic Problem in a Young Woman

**DOI:** 10.4274/balkanmedj.galenos.2020.2019.12.14

**Published:** 2020-06-01

**Authors:** Remzi Tınazlı, Pınar Tunçbilek Özmanevra, Eda Tuna Yalçınozan, Ferhat Erişir

**Affiliations:** 1 Department of Otorhinolaryngology-head and Neck Surgery, Near East University School of Medicine, Nicosia, Cyprus; 2 Department of Otorhinolaryngology-head and Neck Surgery, Kyrenia University School of Medicine, Kyrenia, Cyprus

A 32-year-old woman was referred to our ENT clinics for the evaluation of multiple symmetrical swellings in her neck. In the patient’s family history, there was cross-cousin marriage between her grandparents. The patient and her sister had severe hearing loss and type 1 diabetes mellitus since their childhood. The patient also had symmetric hyperpigmented and erythema nodosum-like lesions on the anterior surface of the lower extremities which would suggest that she has an immune system disorder (Figure 1a). The head and neck examination revealed bilateral symmetrical firm, painless, and fairly mobile periparotid and submandibulary masses (Figure 1b). Magnetic resonance imaging (MRI) scans on the neck revealed conglomerated lymph nodes of a size 45x27 mm on the right parotid region and 37x22 mm on the left and also a solid lymph node of a size 26x18 mm on the right submandibular region and 21x18 on the left. According to the patient’s blood sample tests, C-reactive protein and rythrocyte sedimentation rate were high, while capillary protein electrophoresis showed polyclonal hypergammaglobulinemia. Anti-thyroglobulin, anti-thyroid peroxidase, and anti-nuclear antigen were also positive. The patient’s cardiac MRI showed that there was a uniform contoured mass lesion which is adherent to the interventricular septum with a protrusion through the right ventricular lumen, having the size of 10x15 mm. Compared with the myocardium, it had a hyperintense appearance in the T2-weighted series and a homogenously enhancing mass lesion after IV contrast material injection (Figure 2). Besides, similar mass lesions were observed in the anterior mediastinum. The masses on the neck did not cause any problems other than cosmetic appearance, so excisional biopsy from the parotid and submandibular regions was performed for diagnosis and cosmetic reconstruction. The histopathological evaluation showed that the normal layout of the lymph node was impaired due to the marked enlargement of the lymph sinuses and there are also numerous lymphocytes, plasma cells, and large vesicular nuclei histiocytes within. Most of these histiocytes have intact lymphocytes and plasma cells in their cytoplasm, which is significant for the diagnosis of Rosai-Dorfman disease (RDD), which are referred to as “emperipolesis” or “lymphocytophagocytosis” (Figures 3a and 3b). During the 24-month follow-up period, the size of the mediastinal and intracardiac masses did not change and the skin lesions were not activated. For this report, an informed written consent was obtained from the patient.

Sinus histiocytosis with massive lymphadenopathy, known as RDD, is a rare, nonmalignant histiocytic proliferative disorder. Although the proliferation of the histiocytes is present in the pathogenesis, the etiology is unknown and classically it presents with bilateral, massive, painless cervical lymphadenopathy, but approximately 40% of cases have extranodal involvement. Extranodal RDD mostly involves the skin, nasal cavity and paranasal sinuses, orbita, upper respiratory tract, and bones (1,2). Cardiac involvement is very rare and occurs in less than 0.1% of cases (3). The lesion defined in the heart and mediastinum had a signal intensity similar to what was seen in Daruwalla et al. (4) case. Although the prognosis for RDD is usually good, fatal consequences had to be take into account with the involvement of vital organs such as the heart and mediastinum. Therefore, multidisciplinary researches should be conducted thoroughly.

## Figures and Tables

**Figure 1:**
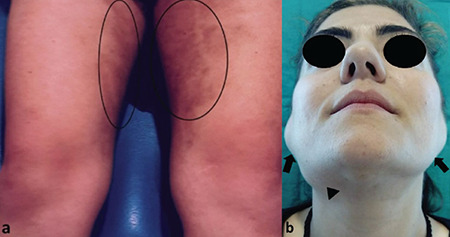
Photos of the patient. (a) The colored skin lesions on her lower extremities. (b) The preoperative view of the patient with the right submandibular (black arrow head) and bilateral parotid region masses (black arrows).

**Figure 2:**
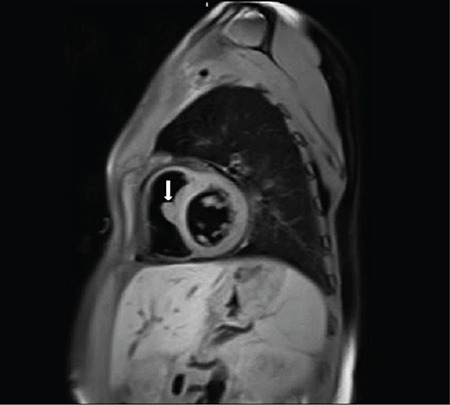
Histophatologic features of the biposy material. Sagittal section with T2 sequences of cardiac magnetic resonance imaging scan indicates a 10x15 mm mass in the cardiac interventricular septum (white arrow).

**Figure 3:**
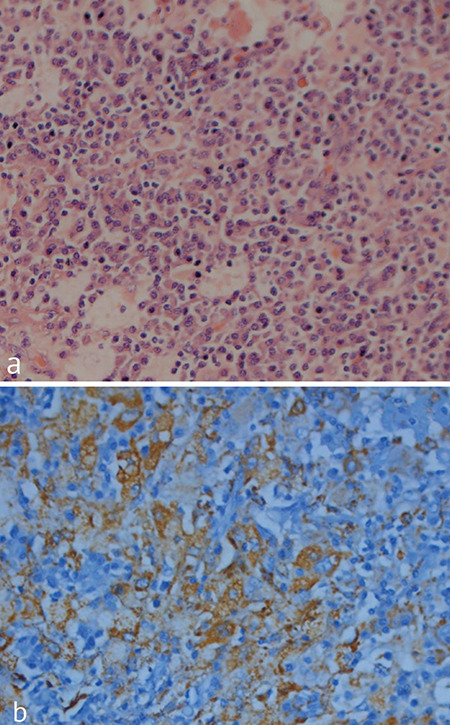
(a) Hematoxylin and eosin staining of the specimen which revealed diffuse aggregats of histiocytoid cells with neutrophils, plasma cells and lymphocytes (HEx100). (b) Emperipolesis of lymphocytes in histiocytes with positivity for immunstain S-100 (x400).
